# *Chilli veinal mottle virus* HCPro interacts with catalase to facilitate virus infection in *Nicotiana tabacum*

**DOI:** 10.1093/jxb/eraa304

**Published:** 2020-06-28

**Authors:** Ting Yang, Long Qiu, Wanying Huang, Qianyi Xu, Jialing Zou, Qiding Peng, Honghui Lin, Dehui Xi

**Affiliations:** 1 Key Laboratory of Bio-Resource and Eco-Environment of Ministry of Education, College of Life Sciences, Sichuan University, Chengdu, Sichuan, PR China; 2 Cardiff University, UK

**Keywords:** Catalase, *Chilli veinal mottle virus*, HCPro, *Nicotiana tabacum*, suppressor of RNA silencing, systemic necrosis

## Abstract

Plant symptoms are derived from specific interactions between virus and host components. However, little is known about viral or host factors that participate in the establishment of systemic necrosis. Here, we showed that helper component proteinase (HCPro), encoded by *Chilli veinal mottle virus* (ChiVMV), could directly interact with catalase 1 (CAT1) and catalase 3 (CAT3) in the cytoplasm of tobacco (*Nicotiana tabacum*) plants to facilitate viral infection. *In vitro*, the activities of CAT1 and CAT3 were inhibited by the interaction between HCPro and CATs. The C-terminus of HCPro was essential for their interaction and was also required for the decrease of enzyme activities. Interestingly, the mRNA and protein level of CATs were up-regulated in tobacco plants in response to ChiVMV infection. *Nicotiana tabacum* plants with HCPro overexpression or *CAT1* knockout were more susceptible to ChiVMV infection, which was similar to the case of H_2_O_2_-pre-treated plants, and the overexpression of CAT1 inhibited ChiVMV accumulation. Also, neither CAT1 nor CAT3 could affect the RNA silencing suppression (RSS) activity of HCPro. Our results showed that the interaction between HCPro and CATs promoted the development of plant systemic necrosis, revealing a novel role for HCPro in virus infection and pathogenicity.

## Teaser

This study uncovered a novel role for HCPro in ChiVMV pathogenicity by interacting with catalases and inhibiting their activities to counter plant immunity.

## Introduction

Due to their sessile nature, plants face various pathogen infections and numerous abiotic stresses during their life cycle. Virus infection is a major problem that affects plant development and causes substantial losses in yield and quality in crops (Fang *et al.*, 2001; Wang, 2015; Jin *et al.*, 2016). To survive environmental changes, plants have evolved a range of defense mechanisms to increase their tolerance. RNA silencing is a well-established plant antiviral response triggered by viral dsRNAs during virus replication in host plants (Hamilton *et al.*, 1999; Niehl *et al.*, 2016). However, many successful viruses have consequently evolved viral suppressors of RNA silencing (VSRs) as strategies to counteract antiviral RNA silencing (Wu *et al.*, 2010; Pumplin and Voinnet 2013).

*Chilli veinal mottle virus* (ChiVMV) is a member of the genus *Potyvirus* in the family *Potyviridae*. ChiVMV infection greatly inhibits plant growth and causes severe symptoms including mottling, distortion, and systemic necrosis (Chung *et al.*, 2008). The ChiVMV genome is a positive-sense, ssRNA of 9.7 kb, excluding the poly(A) tail (Vijayapalani *et al.*, 2012; Gao *et al.*, 2016). Potyviral helper component proteinase (HCPro) is a multifunctional protein mainly involved in aphid transmission and suppression of post-transcriptional gene silencing (Anandalakshmi *et al.*, 1998; Kasschau *et al.*, 1998). In addition, HCPro also acts as a symptom determinant (Kasschau *et al.*, 1998; Hasiów-Jaroszewska *et al.*, 2014). Previous study has proved that the central region and C-terminus are required for RNA silencing suppression (RSS) activity (Urcuqui-Inchima *et al.*, 2000; Varrelmann *et al.*, 2007).

Environmental stresses, such as insufficient water supply, excessive salt, and pathogen attack, could induce the production of reactive oxygen species (ROS) by NADPH oxidases which are encoded by *respiratory burst oxidase homolog* (*Rboh*) genes in plants (Foreman *et al.*, 2003; Gechev *et al.*, 2005; Miller *et al.*, 2009). Accumulation of ROS in cellular compartments affects the cellular redox state and results in oxidative stress. Moreover, ROS also plays a crucial role in sustaining cell growth and inducing hypersensitive cell death in response to a variety of stresses (Mittler *et al.*, 2004). Therefore, tight control of ROS homeostasis is critical (Suzuki *et al*., 2011). Accumulation of ROS is eliminated by antioxidants and scavenging enzymes such as catalase (CAT). CAT, which breaks down H_2_O_2_, is an enzyme found in nearly all living organisms (Chelikani *et al.*, 2004; Gechev *et al.*, 2005).

CATs, which are important participants in the plant antioxidative system (Willekens *et al.*, 1997), are highly expressed enzymes, particularly in certain plant cell types. The tobacco genome encodes three CAT proteins which consist of 492 amino acids and share high sequence similarity (Willekens *et al.*, 1995). CAT1 belongs to Class I CATs which are strongly expressed in photosynthetic tissues, while CAT2 belongs to Class II CATs associated with vascular tissues. CAT3 belongs to Class III CATs that are notably expressed in seeds and reproductive tissues (Willekens *et al.*, 1995). Available evidence from expression patterns and functional analysis suggests that Arabidopsis CAT1, CAT2, and CAT3 correspond to Class III, Class I, and Class II CATs, respectively (Zimmermann *et al.*, 2006; Mhamdi *et al.*, 2010). CAT2 of *Arabidopsis thaliana* was confirmed to be involved in plant defenses (Yuan *et al.*, 2017).

Our previous work showed that ChiVMV infection could cause systemic necrosis in tobacco (Yang *et al.*, 2018). In the present study, we demonstrated that CAT1 and CAT3 could interact with ChiVMV HCPro both *in vitro* and *in vivo*, and determined the critical domain for the interaction between ChiVMV HCPro and CATs. The specific interaction led to attenuation of the CAT activity of host plants but did not affect the RSS activity of ChiVMV HCPro. The accumulation of ChiVMV increased in *CAT1* knockout or HCPro-overexpressing plants, but decreased in CAT1-overexpressing plants. The severity of systemic necrosis of tobacco plants was positively corelated to the accumulation of ChiVMV, indicating that the interaction between ChiVMV HCPro and CATs may be important for virus infection and pathogenicity.

## Materials and methods

### Plant materials and virus inoculation

Plants of wild-type *Nicotiana tabacum* ‘NC89’ (*N. tabacum*, WT), mutants, and transgenic plants were grown in a greenhouse with a 12 h light/12 h dark cycle (100 μmol m^–2^ s^–1^) at 23–25 °C. Six-week-old seedlings were mechanically inoculated with ChiVMV, and phosphate-buffered saline (PBS) rubbed onto the leaves was used as the mock treatment.

### H_2_O_2_ treatment

H_2_O_2_ was purchased from Sigma Aldrich (http://www.sigmaaldrich.com). The concentration of H_2_O_2_ was 100 μM. H_2_O_2_ was sprayed onto *N. tabacum* leaves for 2 d before ChiVMV inoculation. Distilled water was used as a control treatment.

### Superoxide and H_2_O_2_ staining

*Nicotiana tabacum* leaves were vacuum infiltrated with nitro blue tetrazolium (NBT; 0.5 mg ml^–1^) solution for 3 h or 3,3'-diaminobenzidine (DAB; 2 mg ml^–1^) solutions for 8 h for superoxide and H_2_O_2_ staining, respectively. Leaves were then decolorized in boiling ethanol (90%) for 30 min.

### Oxidative damage estimation and chlorophyll fluorescence assay

Leaf relative water content (RWC) was defined as: RWC=(FW–DW)/(TW–DW)×100%, where TW represents turgid leaf weight. Lipid peroxidation was estimated by measuring malondialdehyde (MDA). Detection of the H_2_O_2_ and MDA content was performed by using an H_2_O_2_ and MDA kit (Nanjing Jiancheng, China) and following the manufacturer’s instructions. For the determination of NADPH oxidase activity, the NADPH-dependent superoxide-generating activity was determined as described previously (Deng *et al.*, 2015). The maximum photochemical efficiency of PSII in the dark-adapted state (*F*_v_*/F*_m_) was measured using an Imaging-PAM Chlorophyll Fluorometer equipped with a computer-operated PAM control unit (IMAG-MAXI; Heinz Walz), as described previously (Deng *et al.*, 2015). Three replicates of individual leaves were used for each treatment.

### Plasmid construction and plant transformation

The entire ORFs of *HCPro*, *CAT1*, and *CAT3* were amplified by reverse transcription–PCR and then introduced into the pCM1307 vector to create pCM1307-HCPro-HA, pCM1307-CAT1-HA, and pCM1307-CAT3-HA. CRISPR/Cas9 [clustered regularly interspaced palindromic repeats (CRISPR)/CRISPR-associated protein 9] was used to create the *CAT* knockout, which was constructed as previously described (Wang *et al.*, 2015). The resulting constructs were used for transformation via *Agrobacterium tumefaciens* strain GV3101 (DoubleHelix, Wuhan, China). The overexpressed transgenic lines were selected on half-strength Murashige and Skoog (MS) medium that contained 35 μg ml^–1^ hygromycin. The transgenic lines were analyzed by western blot and quantitative real-time PCR (qPCR). To analyze mutations of CAT, fragments surrounding the target sites of *CAT1* and *CAT3* were amplified by PCR using gene-specific primers *CAT1 F/R*, *CAT3 F1/R1*, and *CAT3 F2/R2*, respectively. Purified PCR products were submitted for direct sequencing with primers.

### Protein expression, purification, and enzyme activity assays *in vitro*

*HCPro*, *CAT1*, *CAT2*, and *CAT3* were amplified by PCR and then inserted into the pGEX-glutathione *S*-transferase (GST) tag and pMAL-C2X maltose-binding protein (MBP) tag. The constructs were transformed into *Escherichia coli* BL21 cells and purified. Indirect assays of CAT activity were performed using a kit (Nanjing Jiancheng, China) according to the manufacturer’s protocol. Mixtures containing 500 pmol CAT and various amounts of HCPro, HC-N, and HC-C (MBP) in a total volume of 100 μl were pre-incubated at 37 °C for 20 min. Mixtures or crude extracta of plant CATs were then added to the working color solution and reacted for 5 min; stop buffer was added to terminate the reaction. Absorbance at 405 nm was measured and activity was calculated.

### Yeast two-hybrid assays

For yeast two-hybrid assays, the full-length coding sequence (CDS) of HCPro was amplified and cloned into pGADT7 (Clontech). The full-length CDSs of CAT1, CAT2, and CAT3 were amplified and cloned into pGBKT7 (Clontech). The yeast strain (AH109) was transformed with pairs of plasmids and grown on Double DO supplement (SD-Leu/-Trp) for 3 d, then the co-transformants were shifted onto Quadruple DO supplement (SD-Leu/-Trp/-Ade/-His) to test for possible interactions.

### Bimolecular fluorescence complementation (BiFC) assays and confocal microscopy

For BiFC assays, the full-length CDS of HCPro was cloned into the pXY104-cYFP vector (Yu *et al.*, 2008). The full-length CDSs of CAT1, CAT2, and CAT3 were each cloned into the pXY103-nYFP vector (Yu *et al.*, 2008). The constructs were transformed into *A. tumefaciens* strain GV3101, and mixed 1:1 immediately prior to being co-infiltrated into *Nicotiana benthamiana* leaves. The transfected plants were grown in the greenhouse for at least 48 h, and fluorescent signals were observed by a scanning microsystem (Leica). Confocal microscopy was performed using a Leica TCS SP5 II system confocal microscope. Green fluorescent protein (GFP) and mCherry were visualized at 488 nm and 543 nm, respectively.

### GST pull-down assays

The GST pull-down assays were performed as described previously (Zhang *et al.*, 2017). Approximately 10 μg of purified GST fusion proteins and GST were incubated with CATs fused to MBP in 500 μl of incubation buffer (50 mM Tris–HCl, pH 6.8, 300 mM NaCl, 1.5% glycerol, 0.6% Triton-X 100, 0.1% Tween) for 2 h at 4 °C. The beads were washed five times with incubation buffer. The washed beads were boiled in 2× SDS loading buffer, and proteins were separated by SDS–PAGE for protein gel blot analysis with anti-GST and anti-MBP antibodies.

### Agroinfiltration and GFP imaging

VSR detection was performed as previously described (Zhang *et al.*, 2018). Equal volumes of *A. tumefaciens* cultures (OD_600_=0.8) harboring plasmids expressing positive sense GFP (sGFP) (Bragg and Jackson, 2004) and *A. tumefaciens* cultures (OD_600_=0.8) harboring pCM1307-HCPro-HA, pCM1307-CAT1-HA, and pCM1307-CAT3-HA expression vectors or an empty vector (EV; pCM1307-HA) control were mixed and co-infiltrated into the leaves of 4- to 5-week-old *N. benthamiana*. GFP fluorescence in the agro-infiltrated plants was photographed under UV light using a long-wave UV lamp (https://www.crystaledge.com/detail/SLXEAxxx). All experiments were repeated three times.

### RNA extraction and qPCR analysis

Total RNA extraction, cDNA synthesis, and qPCR were performed as described by Zhang *et al.* (2010). qPCR analysis was carried out using the SYBR^®^ Premix Ex Taq™ II (TAKARA) on a BIO-RAD CFX Connect™ Real-Time System, following the manufacturer’s instruction. Three independent experiments were performed, and three technical replicates of each experiment were performed. The *Elongation factor 1a* (*EF1a*) gene was used as an internal control for normalization of transcript levels. All primers used for gene expression analysis are shown in Supplementarty Table S1 at *JXB* online.

### Protein extraction and western blotting analysis

Plant material was ground in an Eppendorf tube using 2× SDS sample buffer, centrifuged at 13 000 *g* for 10 min, and the supernatant was saved. For immunoblot analysis, total protein was separated by 10% SDS–PAGE and transferred to polyvinylidene fluoride (PVDF) membranes. The membrane was blocked for 1 h in TBST buffer (10 mM Tris, pH 7.6, 150 mM NaCl, 1.0% Tween-20) with 5% skim milk powder at room temperature and then incubated with specific primary antibodies in TBST buffer for 1 h. After that, the membrane was washed with TBST buffer several times, and the blot was incubated with horseradish peroxide-conjugated secondary antibody (goat anti-rabbit IgG, Thermo Fisher) at a dilution of 1/10 000 for detection by the enhanced chemiluminescence assay.

### Statistical analysis

Samples were analyzed in triplicate, and the data are expressed as the mean ±SD unless noted otherwise. Statistical significance was determined using two-way ANOVA (LSD multiple-range test) or Student’s *t*-test. A difference at *P*<0.05 was considered significant.

## Results

### ChiVMV infection led to systemic necrosis in *N. tabacum*

**Fig. 1.**
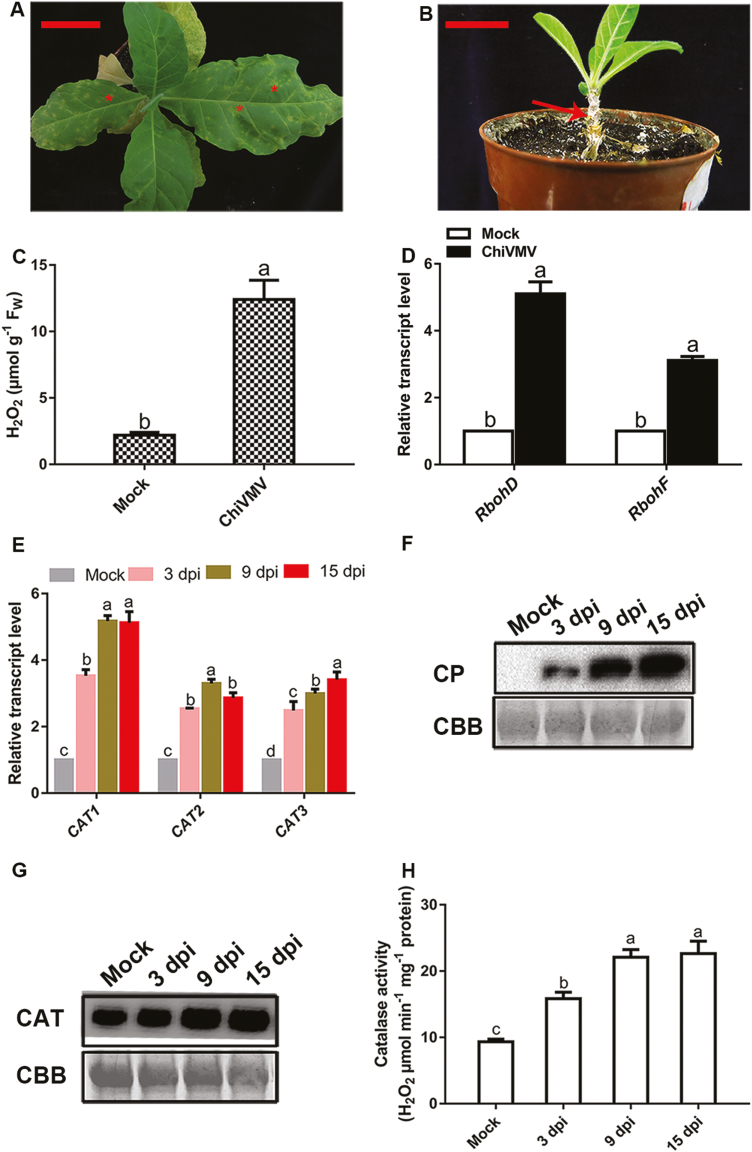
The symptoms of ChiVMV infection on *N. tabacum.* Symptoms of ChiVMV-infected plants at (A) 20 dpi and (B) 40 dpi. Scale bars=5 cm (left panel) and 2.5 cm (right panel). The asterisk indicates the necrotic spot on the leaf and the arrow points to necrosis of the stem. (C) The content of H_2_O_2_ was measured at 9 dpi. (D) qPCR analysis of *RbohD* and *RbohF* expression levels at 9 dpi. (E) The transcript levels of catalase genes at 3, 9, and 15 dpi. (F) Coat protein (CP) of ChiVMV accumulated at 3, 9, and 15 dpi. (G) Western blotting analysis of catalase protein in leaves at 3, 9, and 15 dpi. (H) Catalase activity was measured in mock- and ChiVMV-infected plants. Rubisco proteins were used as loading controls, and were stained using Coomassie brilliant blue (CBB). Systemically infected leaves were collected for detection. Values are means and SDs from three biological replicates per genotype and time point. Lower case letters indicate statistically significant differences (*P*<0.05).

After the *N. tabacum* plants were inoculated with ChiVMV, they developed symptoms of mottling and distortion on the upper leaves at the early infection stages. Subsequently, necrotic lesions appeared throughout all systemic leaves and were sometimes observed on the stem (Fig. 1A, B). Finally, the plant dies with spread of ChiVMV (Fig. 1B). The necrotic spots induced by ChiVMV infection were found to be associated with an increase in H_2_O_2_ production, as measured by H_2_O_2_ content and transcript levels of *RbohD* and *RbohF*, which were a crucial source of H_2_O_2_ (Fig. 1C, D). The results showed that the ChiVMV infection induced ROS accumulation.

### ChiVMV infection altered the expression levels of CATs in *N. tabacum*

H_2_O_2_ is mainly decomposed by CAT in plants; thus we investigated whether ChiVMV infection influences the expression of *CAT* genes and the activity of CAT proteins. Both mock- and ChiVMV-inoculated tobacco leaves were collected at 3 days post-inoculation (dpi), 9 dpi, and 15 dpi. Then, the expression levels of *CAT1*, *CAT2*, and *CAT3* in the collected tissues were determined by qPCR and western blot. As shown in Fig. 1E, *CAT1*, *CAT2*, and *CAT3* mRNAs were induced in ChiVMV-inoculated leaves compared with mock-inoculated leaves. Systemically infected leaves also showed typical symptoms with a high level of ChiVMV accumulation (Fig. 1F). Western blotting with extracts of the same infected leaves also revealed that CAT proteins were accumulated in ChiVMV-inoculated leaves compared with mock-inoculated leaves (Fig. 1G). In addition, the activities of CAT increased under ChiVMV infection (Fig. 1H). Taken together, these results indicated that ChiVMV infection significantly up-regulated the mRNA levels of *CAT1*, *CAT2*, and *CAT3* and the enzymatic activities of their products in host plants.

### HCPro physically interacted with CAT1 and CAT3 *in vitro* and i*n vivo*

**Fig. 2.**
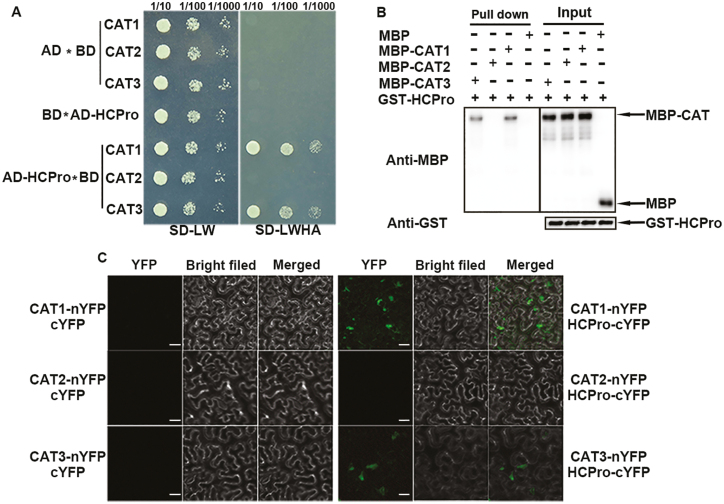
ChiVMV HCPro directly interacts with CAT1 and CAT3 *in vitro* and *in vivo*. (A) Yeast two-hybrid assay. The ability of cells to grow on synthetic dropout medium lacking Leu, Trp, His, and Ade (-LWHA) suggested the interaction. (B) GST pull-down assay showing the interaction among HCPro, CAT1, and CAT3 *in vitro*. Purified CAT1–MBP, CAT2–MBP, CAT3–MBP, or MBP was incubated with HCPro–GST. After being immunoprecipitated with GST beads, the proteins were detected by protein gel blot analysis with anti-MBP or anti-GST antibodies. (C) BiFC assay. HCPro interacted with CAT1 and CAT3 in *N. benthamiana* leaves.Scale bars=30 μm.

To explore in what way CATs were involved in ChiVMV infection, CAT1, CAT2, and CAT3 were used as bait to screen for their possible interaction factor. HCPro was identified as a CAT-interacting protein (Supplementary Fig. S1). Directed yeast two-hybrid assays validated that HCPro interacted with CAT1 and CAT3 but not with CAT2 (Fig. 2A). To further examine whether CAT1, CAT2, and CAT3 directly interacted with HCPro, we performed GST pull-down assays. The results showed that GST–HCPro directly interacted with CAT1 and CAT3 *in vitro*, but not with MBP alone (control) or CAT2–MBP (Fig. 2B). To verify whether HCPro interacted with CAT *in vivo*, the BiFC assay was performed. When CAT1–nYFP (the N-terminus of yellow fluorescent protein) and CAT3–nYFP were co-infiltrated with HCPro–cYFP (the C-terminus of YFP) in *N. benthamiana* leaves, strong YFP fluorescence was observed in the cytoplasm (Fig. 2C). However, leaves expressing CAT2–nYFP and HCPro–cYFP or other control combinations failed to show YFP signals (Fig. 2C, left panel and middle right panel). These results suggested that CAT1 and CAT3 could interact with HCPro *in vivo*.

**Fig. 3.**
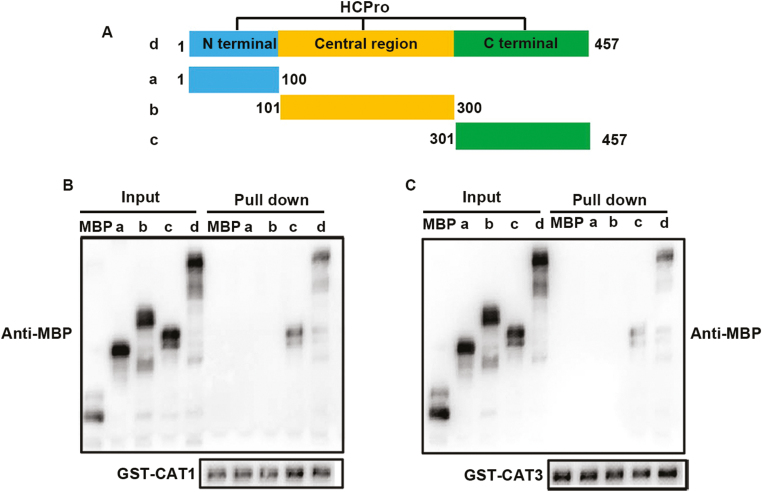
Identification of HCPro domains responsible for the interaction between host factors and HCPro. (A) Schematic description of deletion mutants of ChiVMV HCPro. HCPro can be divided schematically into three regions: the N-terminus (residues 1–100), central region (residues 101–300), and C-terminus (residues 301–457). (B) Identification of the interaction specificity between CAT1 and HCPro from different sources. (C) The C-terminus of HCPro is necessary for the interaction. Different purified deletion mutants of HCPro–MBP or MBP were incubated with CAT1–GST or CAT3–GST. After being immunoprecipitated with GST beads, the proteins were detected by protein gel blot analysis with anti-MBP or anti-GST antibodies.

To investigate which domain of HCPro is necessary for the interaction with CAT1 and CAT3, the CDSs of each functional domain of HCPro reported by Plisson *et al.* (2003) were constructed into vector with an MBP tag (Fig. 3A). As shown in Fig. 3B and C, only the C-terminus of HCPro (HCPro-C, amino acids 301–457) could interact with CAT1 and CAT3 in GST pull-down assays, whereas all other derivatives of HCPro lost their interaction activities, suggesting that the C-terminal fragment of HCPro was required for the interaction.

To further explore the relationship among CAT1, CAT3, and HCPro, we performed co-localization assays. *Agrobacterium* cells expressing HCPro–GFP+CAT1–mCherry, CAT3–mCherry+HCPro–GFP, and the negative controls were co-infiltrated into *N. benthamiana* leaves, followed by confocal microscope observation of their localizations at 3 dpi. Aggregates were observed in plant cells co-expressing HCPro–GFP+CAT1–mCherry and HCPro–GFP+CAT3–mCherry in the cytoplasm, while no aggregate was observed in cells expressing the negative controls (Supplementary Fig. S2). At the same time, we observed the co-localization of CAT1–mCherry, CAT3–mCherry, and HCPro–GFP, and they retained their original localizations in the cytoplasm.

### ChiVMV HCPro inhibited catalase activity via specific protein interactions

**Fig. 4.**
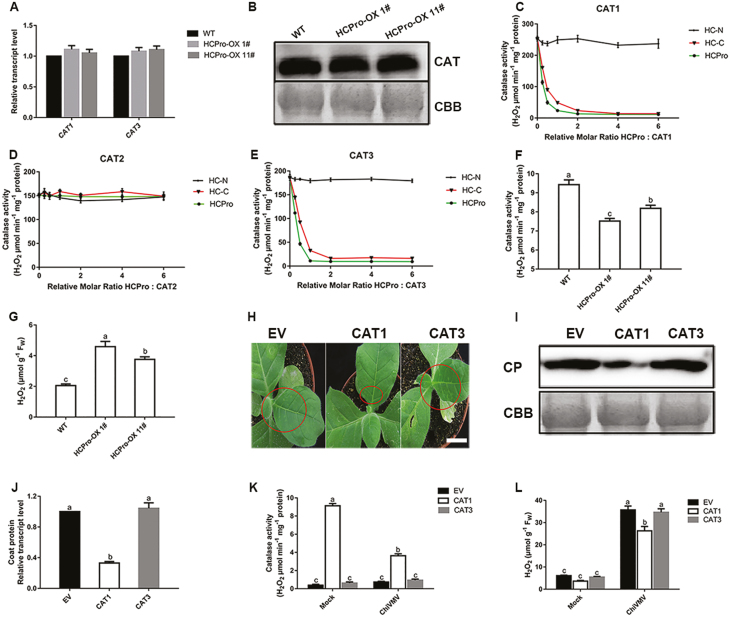
HCPro interacted with CAT1 and CAT3 and inhibited their activities. (A) Gene expression of *CAT1* and *CAT3*, and (B) their protein accumulations in the HCPro-OX lines and WT plants (using 8-week-old seedlings). (C) CAT1, (D) CAT2, and (E) CAT3 enzyme activity assays in reactions containing varying molar ratios of MBP–CAT to deletion mutants of HCPro. CAT (500 pM) was incubated with or without HCPro deletion mutants at 37 °C for the indicated times. MBP was used as negative control. The full length of HCPro is marked as HCPro. HC-N, amino acids 1–100; HC-C, amino acids 301–457. (F) Catalase activity and (G) H_2_O_2_ content in the HCPro-OX lines and WT plants (using 8-week-old seedlings). (H) Necrosis symptoms, (I) coat protein (CP) accumulation, and (J) virus replication of EV, CAT1, and CAT3 introduced into *cat1cat3*-KO plants under ChiVMV infection. The circles indicate the area of necrosis. Scale bars=2.5 cm. (K) Catalase activity and (L) H_2_O_2_ content in EV, CAT1, and CAT3 introduced into *cat1cat3*-KO plants. Fw, fresh weight; EV, empty vector. Values are means and SDs from three biological replicates per genotype and time point. Lower case letters indicate statistically significant differences (*P*<0.05).

To evaluate the biological significance of this specific interaction, HCPro transgenic *N. tabacum* lines (HCPro-OX) were generated (Supplementary Fig. S4) to test the levels of CAT1 and CAT3. The results showed that the levels of *CAT1* and *CAT3* mRNA and CAT protein did not change in response to HCPro (Fig. 4A, B). Then CAT1, CAT2, CAT3, and various truncated versions of HCPro were fused to MBP and expressed in *E. coli* BL21 cells, and the enzymatic activities of CATs *in vitro* were analyzed. CAT1, CAT2, and CAT3 were pre-incubated for 20 min with varying amounts of various truncated versions of HCPro (the molar ratio of the truncated versions of HCPro:CATs ranged from 0 to 6-fold ) (Supplementary Fig. S3). As shown in Fig. 4C, the enzymatic activity of CAT1 was inhibited by HC-C and HCPro with an increased molar ratio of truncated versions of HCPro:CAT1 from 0–2, but a further increased molar ratio had no significant effects on CAT1 activity. In the reaction of HC-N:CAT1 with the same molar ratio, HC-N did not affect CAT1 activity. In addition, truncated versions of HCPro and the full length of HCPro did not affect CAT2 enzymatic activity (Fig. 4D). However, CAT3 enzymatic reactions to which HC-C or HCPro protein were added appeared to have a lower level of CAT activity compared with that to which HC-N protein was added (Fig. 4E). The results demonstrated that HCPro interacted with CAT1 and CAT3 through their C-terminus and inhibited their activities *in vitro*.

To further illustrate the effects of the interaction between HCPro and CAT, we quantified the CAT activities in HCPro-OX plants. The results showed that CAT activity was inhibited in HCPro-OX plants compared with WT plants (Fig. 4F). Furthermore, the content of H_2_O_2_ was lower in WT plants than in HCPro-OX plants (Fig. 4G). To further confirm whether the H_2_O_2_ level was affected by interactions among CAT1, CAT3, and HCPro, the EV, CAT1, and CAT3 were introduced into the double *cat1cat3* knockout (KO) lines to generate transiently expressing lines. As shown in Fig. 4K, the CAT activity was reduced in CAT1 transient expression plants under ChiVMV infection compared with mock-inoculated plants. However, leaves infiltrated with EV or CAT3 transient expression plants exhibited no significant difference in CAT activity compared with mock-inoculated leaves. As for H_2_O_2_ levels, no significant differences were detected in ChiVMV-inoculated EV and CAT3 plants. However, the H_2_O_2_ concentration was higher in ChiVMV-infected CAT1 transient expression plants than in mock-inoculated plants but lower than in ChiVMV-infected EV or CAT3 transiently expressing plants (Fig. 4L). In an additional experiment, EV and CAT3 plants developed more serious symptoms and showed higher accumulations of virus compared with CAT1 plants (Fig. 4H–J). These results suggested that CAT1 activity was inhibited by the interaction between CAT1 and HCPro in *N. tabacum*.

### CAT1 played an antiviral role during ChiVMV infection

**Fig. 5.**
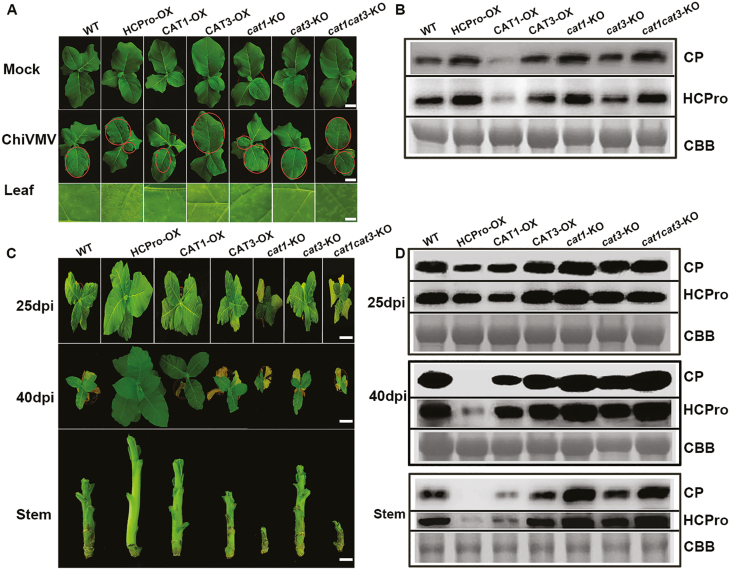
Overexpression of HCPro and knockout of *CAT1* enhanced ChiVMV infection, whereas knockout of *CAT3* did not affect ChiVMV infection. (A and C) Symptoms of the mock-inoculated or ChiVMV-infected WT and transgenic plants at 9, 15, and 40 dpi. Scale bars=5 cm (upper panel) and 0.25 cm (lower panel) in (A). Scale bars=10 cm (upper panel), 20 cm (middle panel), 1.5 cm (lower panel) in (C). (B and D) Detection of ChiVMV HCPro and coat protein (CP) in ChiVMV-infected WT and transgenic plants by western blot at 9, 25, and 40 dpi. Systemically infected leaves were collected for detection.

To investigate whether CAT1, CAT3, or HCPro could alter ChiVMV infection, overexpressing transgenic lines of CAT1, CAT3 (CAT1-OX and CAT3-OX), and HCPro-OX, and knockout transgenic lines of *cat1*, *cat3*, and double *cat1cat3* (*cat1*-KO, *cat3*-KO, and *cat1cat3*-KO) were generated (Supplementary Figs S4, S5). As shown in Supplementary Fig. S6, the transgenic plants had no obvious phenotype, except for HCPro-OX which displayed a long, narrow petiole and a slight curling of the leaves at 5–7 weeks old; however, this phenotype disappeared subsequently. Thirty-six seedings of each line were inoculated with ChiVMV and observed. At 9 dpi, *cat1*-KO, *cat1cat3*-KO, and HCPro-OX plants displayed more serious necrosis than WT plants, while CAT1-OX plants showed mild symptoms, suggesting that *cat1*-KO, *cat1cat3*-KO, and HCPro-OX plants were more susceptible to ChiVMV infection, while the CAT1-OX lines displayed higher tolerance (Fig. 5A). Western blot assays of HCPro and the coat protein of ChiVMV revealed that virus accumulation was higher in *cat1*-KO and HCPro-OX plants than in WT plants, and the accumulation of that in CAT1-OX plants was the least (Fig. 5B). The accumulation of ChiVMV coat protein and HCPro in systemic leaves was detected at 25 and 40 dpi. The results showed that the virus accumulation in *cat1*-KO and *cat1cat3*-KO plants was the highest, followed by that in WT, CAT3-OX, and *cat3*-KO plants, and that in CAT1-OX plants was the lowest (Fig. 5C, D). Unexpectedly, there was no detectable viral protein in newly emerging systemic leaves of virus-infected HCPro-OX plants at the late stage of infection (40 dpi), suggesting that these plants were recovering from the infection (Fig. 5C, D). The accumulation of virus was still at a high level in *cat1*-KO and *cat1cat3*-KO plants, and they showed stunted phenotypes and serious necrosis in the stem. In contrast, CAT1-OX plants showed minimal necrosis in the stem (Fig. 5C). These results indicated that CAT1 functioned as an antiviral compound in response to ChiVMV infection.

### CAT1 alleviated oxidative damage and modulated ROS balance under ChiVMV infection

**Fig. 6.**
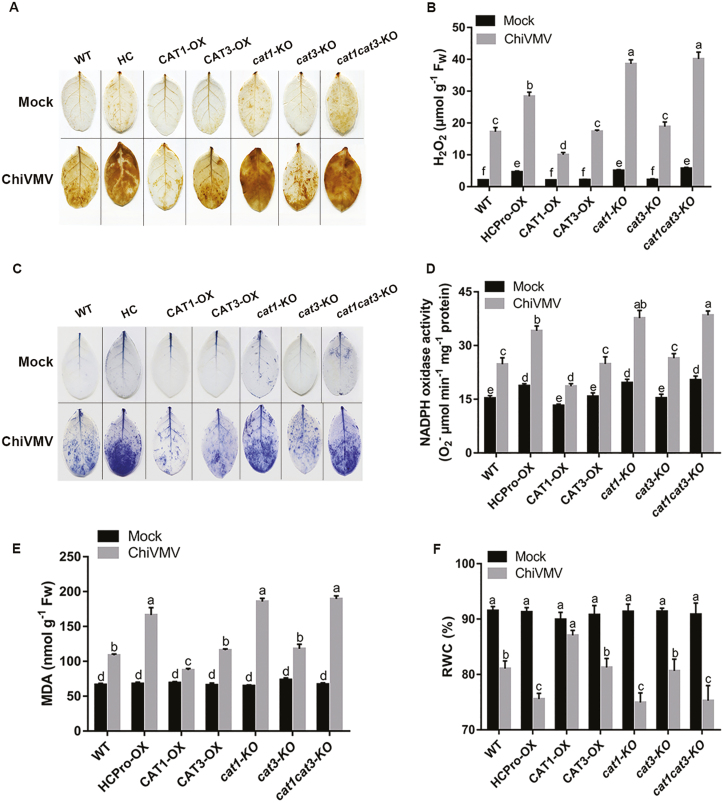
Oxidative damage of plants with or without ChiVMV infection. (A) H_2_O_2_ levels were detected by DAB staining. (B) Quantitative measurements of H_2_O_2_ content. (C) Superoxide contents were detected by NBT staining. (D) Quantitative measurements of NADPH oxidase activity. Quantitative measurements of (E) RWC and (F) MDA content. Systemically infected leaves were collected for detection. Bars represent the mean and SD of values obtained from three biological repeats. Significant differences (*P*<0.05) are denoted by different lower case letters. F_W_, fresh weight.

Plant responses to various types of stress are associated with the generation of ROS (Baxter *et al.*, 2014). We further detected the accumulations of superoxide and H_2_O_2_ using NBT and DAB staining procedures, respectively. Both procedures detected decreased staining in CAT1-OX leaves compared with WT under ChiVMV infection. However, DAB and NBT staining were greatly increased in *cat1*-KO and HCPro-OX leaves under virus infection (Fig. 6A, C). We further analyzed H_2_O_2_ content and NADPH oxidase activity. In HCPro-OX, *cat1*-KO, and *cat1cat3*-KO leaves, NADPH oxidase activity and H_2_O_2_ content were significantly higher than in CAT3-OX or *cat3*-KO under ChiVMV infection, while these increases were largely alleviated in CAT1-OX leaves (Fig. 6B, D). MDA content and RWC could also indicate the degree of damage to plants caused by environmental stress. Consistent with the ROS accumulation in Fig. 6A–D, the CAT1-OX plants showed lower levels of MDA, while the *cat1*-KO plants showed enhanced levels of MDA compared with the WT plants (Fig. 6E). In addition, CAT1-OX plants maintained a higher RWC than WT plants under virus infection, while the RWC in HCPro-OX plants decreased significantly (Fig. 6F). Interestingly, the MDA content and RWC in CAT3-OX or -KO plants were similar to those in WT plants (Fig. 6E, F). To confirm the role of CAT3 in response to ChiVMV infection, the CAT activities of the WT and mutants were examined. Consistent with published results, knockout of *CAT1* but not of *CAT3* significantly reduced CAT activity in *N. tabacum* (Supplementary Fig. S7). These results illustrated that CAT1 played a leading role in regulating CAT activity, which alleviated oxidative damage.

### Pre-treatment with H_2_O_2_ increased the susceptibility of plants to ChiVMV

**Fig. 7.**
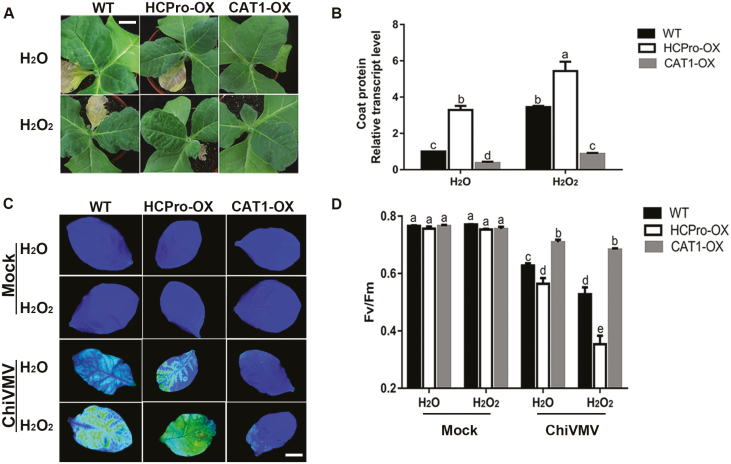
Pre-treatment with H_2_O_2_ increased susceptibility of plants to ChiVMV infection. (A) Symptoms of the ChiVMV-infected WT, HCPro-OX, and CAT1-OX plants with H_2_O_2_ or H_2_O pre-treatment at 9 dpi. Scale bars=2 cm. (B) Detection of virus accumulation in ChiVMV-infected WT, HCPro-OX, and CAT1- OX plants with H_2_O_2_ or H_2_O pre-treatment at 9 dpi by qPCR analysis. (C) Images of the maximum PSII quantum yield (*F*_v_*/F*_m_) in WT, HCPro-OX, and CAT1-OX plants with H_2_O_2_ or H_2_O pre-treatment at 9 dpi. (D) Average values of *F*_v_*/F*_m_ for the respective chlorophyll fluorescence images. Ten plants were used for each treatment, and a picture of one representative leaf is shown. Systemically infected leaves were collected for detection. Bars represent the mean and SD of values obtained from six independent plants. Significant differences (*P*<0.05) are denoted by different lower case letters.

To investigate a possible role for H_2_O_2_ in plant response to ChiVMV, WT, HCPro-OX, and CAT1-OX plants were chosen for further analysis. In H_2_O_2_-pre-treated plants, the necrosis symptoms were more serious than in water-pre-treated plants (Fig. 7A). As shown in Fig. 7B, virus expression in WT plants was increased compared with CAT1-OX plants but reduced compared with HCPro-OX plants under H_2_O_2_ pre-treatment. Changes of photosynthetic parameters of PSII under stress conditions were determined. The results showed that in H_2_O_2_-pre-treated plants, the maximum photochemical efficiency of PSII in the dark-adapted state (*F*_v_*/F*_m_) was significantly lower than that in water-pre-treated plants under ChiVMV infection (Fig. 7C, D). In contrast, the *F*_v_*/F*_m_ of H_2_O_2_- and water-pre-treated mock plants were at the same level (Fig. 7D). These results demonstrated that H_2_O_2_ promoted ChiVMV accumulation, which disturbed the PSII function of tobacco plants.

### Influences of CAT1 and CAT3 on the RSS activity of HCPro

**Fig. 8.**
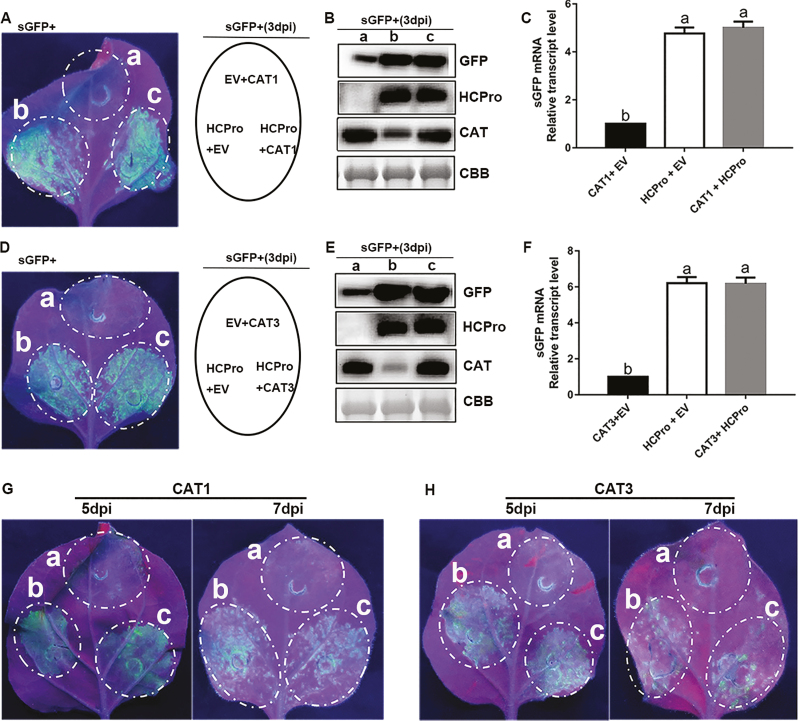
The local RSS activity of ChiVMV HCPro was not influenced by host factors CAT1 and CAT3. (A and D) *Nicotiana benthamiana* leaves were infiltrated with a mixture of three *A. tumefaciens* cultures carrying different constructs, as indicated in the middle panel, and photographed at 3 dpi. (B and E) Western blot analysis of protein extracts from *N. benthamiana* leaves infiltrated with mixtures of *A. tumefaciens* carrying different constructs as indicated on the left. The expression of CAT1 and CAT3 was confirmed with CAT antibody. The expression of HCPro and GFP was confirmed with HCPro and GFP antibodies, respectively. Coomassie Brilliant Blue (CBB) staining of the large subunit of Rubisco was used as a loading control. (C and F) qPCR analysis of the GFP mRNA accumulation level. Bars represent the mean and SD of values obtained from three biological repeats. Significant differences (*P*<0.05) are denoted by different lower case letters. (G and H) *Nicotiana benthamiana* leaves were infiltrated with a mixture of three *A. tumefaciens* cultures and photographed at 5 and 7 dpi.

Given that HCPro has been well characterized as a VSR, the effects of CAT1 and CAT3 on the RSS activity of HCPro were analyzed. To achieve this, *N. benthamiana* leaves were co-infiltrated with a mixture of *A. tumefaciens* carrying pGD-GFP. The results showed that the mixture of HCPro and EV exhibited high fluorescence in the agroinfiltrated patches due to the RSS activity of HCPro (Fig. 8A, D). This was also the case when CAT1 or CAT3 was co-infiltrated with pGD-GFP plus HCPro at 3 dpi (Fig. 8A, D). In contrast, tissues co-infiltrated with pGD-GFP plus CAT1 or CAT3 and EV exhibited very faint green fluorescence (Fig. 8A, D). Western blot analysis showed that GFP levels were positively correlated with the intensity of green fluorescence and the abundance of HCPro protein (Fig. 8B, E). qPCR analysis also showed that GFP mRNA levels correlated directly with the intensity of green fluorescence (Fig. 8C, F). In addition, co-infiltration of CAT and HCPro displayed the same level of green fluorescence as a mixture of HCPro and EV at 5 and 7 dpi (Fig. 8G, H). Taken together, these results suggested that interactions between CATs and HCPro could not attenuate the RSS activity of ChiVMV HCPro.

## Discussion

Rapid production of ROS is associated with diverse physiological processes (Xia *et al.*, 2009; Zhou *et al.*, 2014; Deng *et al.*, 2016). On the one hand, some studies also suggested that low levels of ROS enhance tolerance against various types of stresses (Baxter *et al.*, 2014; Xu *et al.*, 2014; Deng *et al.*, 2016). On the other hand, ROS are harmful byproducts of aerobic metabolism or results of pathogen infection, which induce oxidative burst with elevated levels of H_2_O_2_ (Choi *et al.*, 2007; Deng *et al.*, 2015). CATs are important antioxidative enzymes that contribute to maintain the redox balance and response to various stresses (Mhamdi *et al.*, 2012; Li *et al.*, 2015). Our present study showed that ChiVMV infection caused H_2_O_2_ burst and systemic necrosis in *N. tabacum* (Fig. 1B, D). In addition, the transcript levels of *CAT* genes and CAT activity were increased in response to ChiVMV infection (Fig. 1G, H). Furthermore, WT plants pre-treated with H_2_O_2_ suffered a dramatic increase of virus accumulation and had more serious symptoms (Fig. 7). In contrast, overexpression of CAT1 reduced ChiVMV accumulation and alleviated symptoms in *N. tabacum* plants, whereas knockout of *CAT1* increased the virus accumulation (Fig. 5). These findings suggested that CATs played an antiviral role in *N. tabacum* plants in response to ChiVMV infection.

The effects of interaction between CATs and the virus varied in different plant–pathogen systems. For example, CAT activity was inhibited by indirect interactions between the 2b protein of *Cucumber mosaic virus* and CAT3. Nevertheless, the interaction between triple gene block protein 1 and tomato CAT1 enhanced CAT activity to facilitate virus accumulation (Inaba *et al.*, 2011; Mathioudakis *et al.*, 2013). In this study, we found that HCPro of ChiVMV could interact with CAT1 and CAT3 *in vitro* and *in vivo* to inhibit their activities (Figs 2, 4; Supplementary Fig. S1), and the C-terminal domain of HCPro was critical for the interaction (Fig. 3). The inhibition of CAT activities induced a high level of H_2_O_2_, which was toxic for plants and contributed to virus accumulation. In addition, HCPro-OX plants exhibited reduced CAT activity and accumulated a high level of virus compared with WT plants at the early stage post-viral infection (Figs 4, 5). All of these findings demonstrated that the interaction between HCPro and CAT contributed to virus accumulation.

Certain plant–virus interactions lead to disease recovery at later stages of infection via crosstalk between post-transcriptional gene silencing (PTGS) and transcriptional gene silencing (TGS) pathways involved in the disease recovery (Ratcliff *et al.*, 1997; Baulcombe, 2004; Kørner *et al.*, 2018). A previous study demonstrated that a high expression level of *Potato virus A* HCPro showed a peculiar recovery phenotype in plants due to the level of sequence homology between the virus and the overexpressed gene (Savenkov and Valkonen, 2002). In HCPro-OX lines, interaction between HCPro and CAT1 led to a lower level of CAT activity and resulted in more virus accumulation and serious oxidative damage in HCPro-OX plants than in WT plants at 9 dpi (Figs 4F, 5A, B, 6). However, the necrosis symptoms disappeared in HCPro-OX plants, and the virus concentration was reduced at 25 dpi compared with WT plants. Furthermore, no virus was detectable in leaves and stems, and plants appeared ‘healthy’ in the HCPro-OX line at 40 dpi (Fig. 5C, D), This implied that although the infected HCPro-OX plants showed more serious disease symptoms due to the high level of HCPro inhibiting the activity of CATs at the early stage of virus infection, the RNA silencing pathway based on dsRNA of HCPro played an antiviral role at the late stage of infection, resulting in eventual plant ‘recovery’.

VSRs have been regarded as a counter for plant defenses to facilitate viral infection (Mathioudakis *et al.*, 2013; Chen *et al.*, 2017; Hafrén *et al*., 2018). The VSR of *Tomato yellow leaf curl China virus* increased the expression of the tobacco calmodulin-like protein rgs-CaM to strengthen the activity of RSS (Makiyama *et al.*, 2016). To muzzle virus attack, host factors have also been illustrated to affect the activity of VSR (Wu *et al.*, 2010; Nakahara and Masuta 2014). *Nicotiana tabacum* rgs-CaM was shown to bind to VSR protein and attenuated the RSS activity of VSR against viral infection (Nakahara *et al.*, 2012). Canto *et al.* (2006) and Chen *et al.* (2017) proved that the silencing suppression activities of VSR were altered by a host factor. HCPro of *Potyviridae* is a multitasking protein for viral transmission, polyprotein maturation, and RNA silencing suppression (Valli *et al.*, 2018). Our present study showed that the interaction between CATs and ChiVMV HCPro failed to repress the RSS activity of HCPro (Fig. 8). It also showed that viruses can use various strategies to resist plant defense, and our work demonstrated a novel role for HCPro in virus infection and pathogenicity.

In conclusion, our results indicated that systemic necrosis caused by ChiVMV infection in *N. tabacum* plants was related to the interaction between ChiVMV silencing suppressor protein HCPro and NtCATs. On the one hand, plants developed CAT to remove harmful H_2_O_2_ for maintaining cell homeostasis under stress conditions. As a counter for plant defense, the virus also employed mechanisms such as VSRs to facilitate their life activities. When encountering virus infection, CATs in *N. tabacum* plants were firstly activated in response to ChiVMV infection. Then, HCPro interacted with CAT1 to inhibit CAT activity, resulting in H_2_O_2_ generation to aid virus infection. Finally, ROS burst induced systemic cell death of infected plants. This work demonstrates a novel role for VSRs in virus–host interactions and contributes to our understanding of complex viral counter-host mechanisms.

## Supplementary data

Supplementary data are available at *JXB* online.

Fig. S1. CATs specifically interacted with HCPro.

Fig. S2. CAT1 and CAT3 co-localized to ChiVMV HCPro in *N. benthamiana* leaves.

Fig. S3. Coomassie brilliant blue staining of CAT1, CAT2, CAT3, and deletion mutants of HCPro at the varying amounts used in this assay.

Fig. S4. Identification of overexpression transgenic plants.

Fig. S5. Phenotype and identification of *cat1*-KO, *cat3*-KO, and *cat1cat3*-KO transgenic lines.

Fig. S6. Phenotype of WT and transgenic lines at different growing stage in T_1_ lines.

Fig. S7. Catalase activity of WT and transgenic lines.

Table S1. Primers used for construction of vectors and real-time PCR analysis.

eraa304_suppl_Supplementary_Figures

eraa304_suppl_Supplementary_Table_S1

## Data Availability

All data in this manuscript are fully available without restriction.
