# Supplementary material for: Chilli veinal mottle virus HCPro interacts with catalase to facilitate virus infection in Nicotiana tabacum
Source: J Exp Bot. 2020 Jun 28;71(18):5656–68. doi: 10.1093/jxb/eraa304 (PMC7501817; doi:10.1093/jxb/eraa304)
Supplement: eraa304_suppl_Supplementary_Figures [file eraa304_suppl_supplementary_figures.pdf]

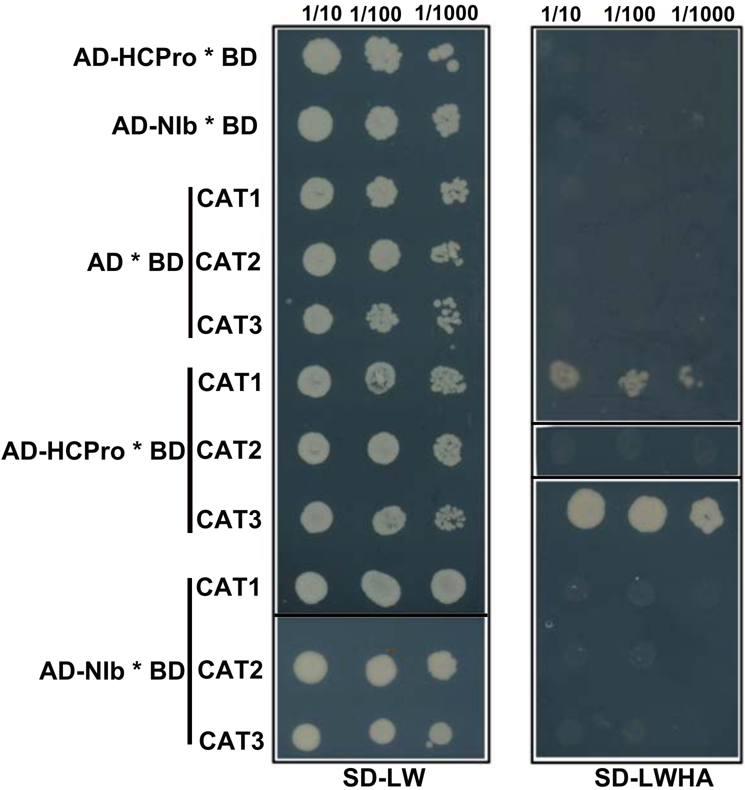

Figure S1. CATs specifically interacted with HCPro.

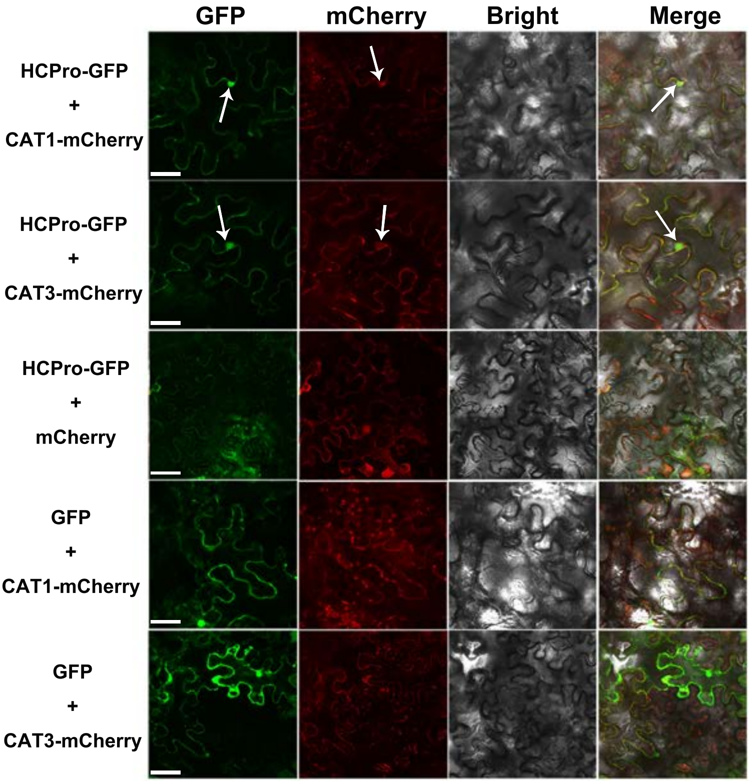

Figure S2. CAT1 and CAT3 colocalized to ChiVMV HCPro in *N. benthamiana* leaves. Scale bars = 50 μm.

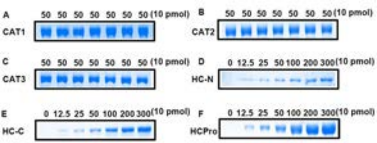

Figure S3. Coomassie brilliant blue staining of CAT1, CAT2, CAT3 and deletion mutants of HCPro at the varying amounts used in this assay. HC-N (1–100 aa), HC-C (301–457 aa). The full length of HCPro is marked as HCPro.

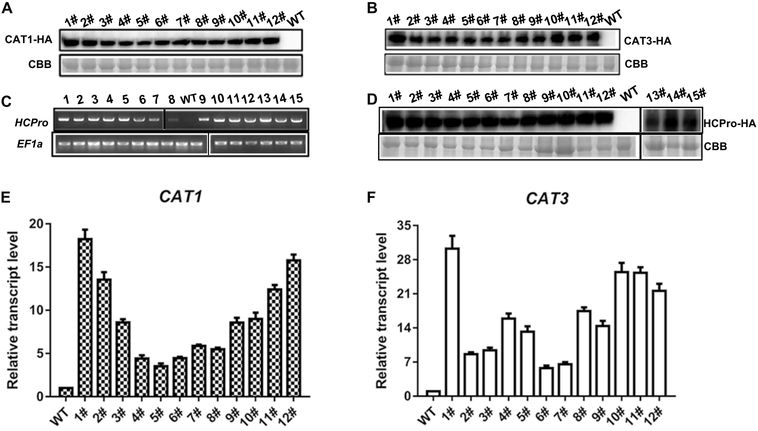

Figure S4. Identification of overexpression transgenic plants.

Western blot analysis of (A) CAT1 expression and (B) CAT3 expression by HA antibody. (C) Analysis of HCPro expression by reverse transcription PCR and (D) Western blot. *EF1a* was used as loading control. qPCR analysis of (E) *CAT1* and (F) *CAT3* expression levels in T0 lines. Expression levels were standardized to *EF1a*, and result of WT was set at 1. Bars represent mean and standard deviation of values obtained from three biological repeats.

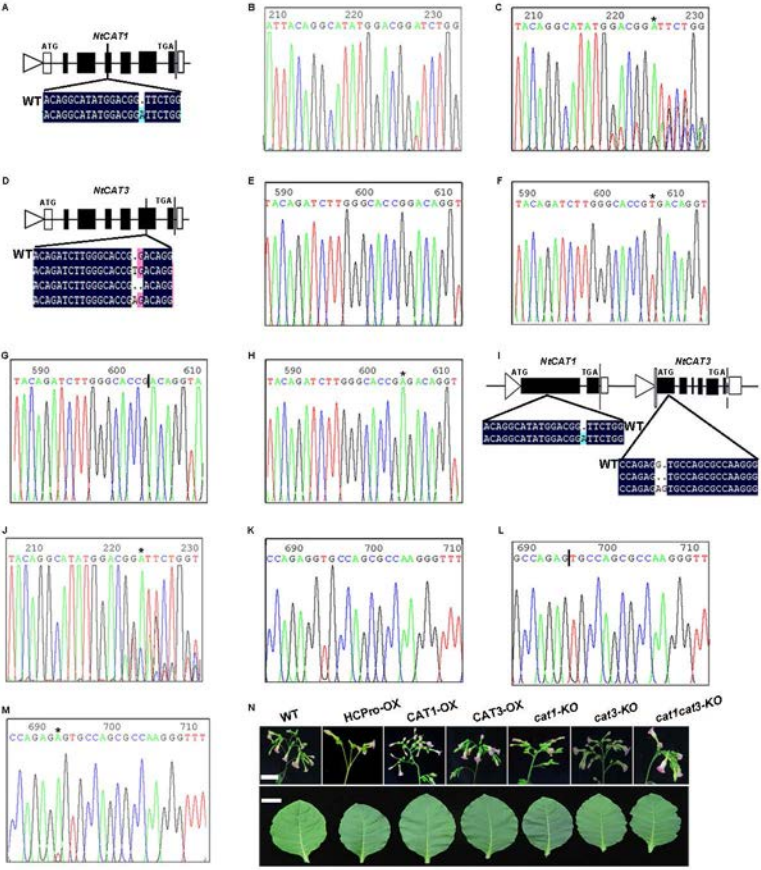

Figure S5. Phenotype and identification of cat1-KO, cat3-KO, cat1cat3-KO transgenic lines. (A) Sequencing analysis of targeted region mutations of *CAT1* in representative T0 lines. Sanger sequencing chromatogram of cloned *CAT1* in (B) WT and (C) *cat1*-KO plants. (D) Sequencing analysis of targeted region mutations of *CAT3* in representative transgenic T0 lines. Sanger sequencing chromatogram of cloned *CAT3* in (E) WT and (F), (G), (H) *cat3*-KO T0 plants. (I) Sequencing analysis of targeted region mutations of *CAT1* and *CAT3* in *cat1cat3-KO* representative T0 lines. (J) Representative Sanger sequencing chromatograms of target sit from the *CAT1* locus in *cat1cat3*-KO lines. Representative Sanger sequencing chromatograms of *CAT3* target sit from the *CAT3* locus (K) in WT and (L), (M) in *cat1cat3*-KO lines. Detected insert base was marked in asterisk and delete base was marked by a vertical line. (N) Phenotypes of WT and transgenic plant in T0 lines. Scale bars = 6 cm (upper panel) and 2.5 cm (lower panel).

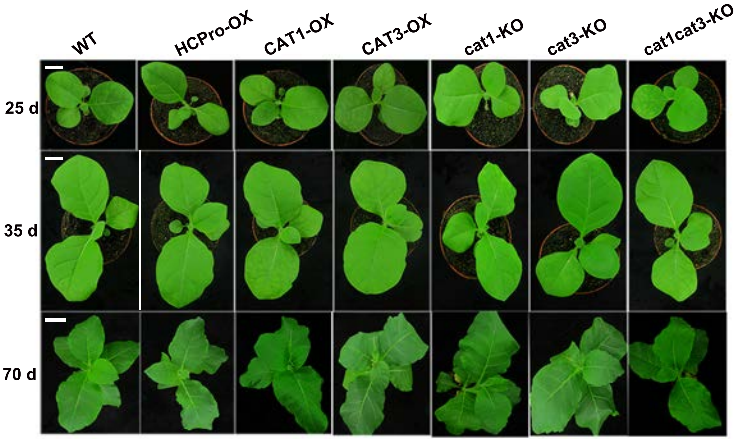

Figure S6. Phenotype of WT and transgenic lines at different growing stage in T1 lines.

Scale bars = 2.5 cm (upper and middle panel) and 8 cm (lower panel).

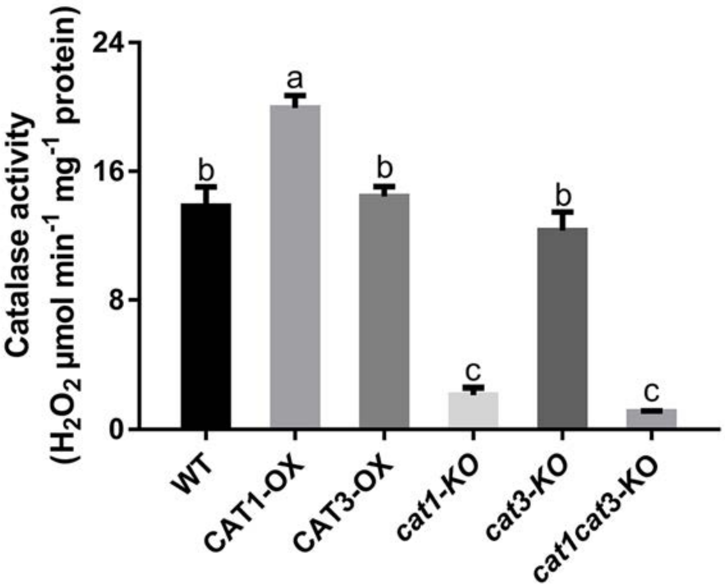

Figure S7. Catalase activity of WT and transgenic lines.
Bars represent mean and standard deviation of values obtained from three biological repeats. Significant differences (P<0.05) are denoted by different lowercase letters.

Primer Names	Primer Sequences (5'-3')	Purpose
Yeast-CAT1-F	CCGGAATTC ATGGCAAGTG AAAAGTGGTT	Y2H
Yeast-CAT1-R	GCACTGCAGTTACTGATGGGTGACTTCTC	Y2H
Yeast-CAT2-F	CCGGAATTCATGGATCCCTCTAAGTTTCGA	Y2H
Yeast-CAT2-R	GCACTGCAGTCACATTGTAGGCTTTAAAG	Y2H
Yeast-CAT3-F	CCGGAATTCATGGATCCATACAAGTATCG	Y2H
Yeast-CAT3-R	CGCGTCGACTCACATTGTGGGCCTTACAT	Y2H
Yeast-HCPro -F	CCGGAATTC ATGTCAGCAGGCGAGCTCTT	Y2H
Yeast-HCPro -R	CGCGGATCCCTAACCAACTCTGTACATT	Y2H
MBP-CAT1-F	CCGGAATTC ATGGCAAGTG AAAAGTGGTT	Pull down
MBP-CAT1-R	GCACTGCAGTTACTGATGGGTGACTTCTC	Pull down
MBP-CAT2-F	CCGGAATTCATGGATCCCTCTAAGTTTCGA	Pull down
MBP-CAT2-R	GCACTGCAGTCACATTGTAGGCTTTAAAG	Pull down
MBP-CAT3-F	CCGGAATTCATGGATCCATACAAGTATCG	Pull down
MBP-CAT3-R	CGCGTCGACTCACATTGTGGGCCTTACAT	Pull down
GST-HCPro-F	CCGGAATTCATGTCAGCAGGCGAGCTCTT	Pull down
GST-HCPro-R	CGCGTCGACCTAACCAACTCTGTACATT	Pull down
GST-CAT1-F	CCGGAATTCATGGCAAGTG AAAAGTGGTT	Pull down
GST-CAT1-R	GCAGTCGACTTACTGATGGGTGACTTCTC	Pull down
GST-CAT3-F	CCGGAATTCATGGATCCATACAAGTATCG	Pull down
GST-CAT3-R	CGCGTCGACTCACATTGTGGGCCTTACAT	Pull down
MBP-HCPro-F	CCGGAATTCATGTCAGCAGGCGAGCTCTT	Pull down
MBP-HCPro-R	CGCGTCGACCTAACCAACTCTGTACATT	Pull down
MBP-HC-N-F	CCGGAATTCATGTCAGCAGGCGAGCTCTT	Pull down
MBP-HC-N-R	CGCGTCGACGCTTTTAAGTAATTGTACAGCA	Pull down

MBP-HC-F	CCGGAATTCATGCACTTTTTAAGCTTTAAA	Pull down
MBP-HC-R	CGCGTCGACCGATCCATCATCATAAGTTACA	Pull down
MBP-HC-C-F	CCGGAATTCATGCCAGTTTTGTCAGAATTCAA	Pull down
MBP-HC-C-R	CGCGGATCCCTAACCAACTCTGTACATT	Pull down
BiFC-CAT1 -F	TGCTCTAGAATGGATCCATACAAGTACCG	BiFC
BiFC-CAT1-R	CGCGTCGACTATGCTTGGTCTCACA	BiFC
BiFC-CAT2-F	TGCTCTAGAATGGATCCCTCTAAGTTTCG	BiFC
BiFC-CAT2-R	CGCGTCGACCATTGTAGGCTTTAAAG	BiFC
BiFC-CAT3-F	TGCTCTAGAATGGATCCATACAAGTATCG	BiFC
BiFC-CAT3-R	CGCGTCGACCATTGTGGGCCTTACAT	BiFC
BiFC-HCPro-F	CCGGAATTCATGTCAGCAGGCGAGCTCTT	BiFC
BiFC-HCPro-R	CGCGTCGACCTAACCAACTCTGTACATT	BiFC
CAT1-F	TCCACAAGATTACAGGCATA	qPCR
CAT1-R	AGCGGCAATAGAGTCATAG	qPCR
CAT2-F	CCAATTCCTTCTCGTGTCT	qPCR
CAT2-R	GTATCTGTCTTGCCTGTCA	qPCR
CAT3-F	AGGAGGAGCGAATCATAGT	qPCR
CAT3-R	TTCAATACCAAGCGACCAA	qPCR
EF1α-F	TGCTGTAACAAGATGGATGC	qPCR
EF1α-R	AGATGGGGACAAAGGGGATT	qPCR
Coat protein-F	AAACCCAGCCACAGTCTCGT	qPCR
Coat protein-R	ATCTCCGTCCATCATCACCC	qPCR
NbEF1α-F	AGCTTTACCTCCCAAGTCATC	qPCR
NbEF1α-R	AGAACGCCTGTCAATCTTGG	qPCR
GFP-F	ACATTATGGCAGACAAACAA	qPCR

GFP-R	TTACAAACTCAAGAAGCACC	qPCR
HCPro - F	ATGTCAGCAGGCGAGCTCTT	RT-PCR
HCPro - R	CTAACCAACTCTGTACATT	RT-PCR
RbohD-F	ACACGATCACATGGCTTCGAAAT	Pull down
RbohD-R	GTGTATTCCAACCCCAAGAGCA	Pull down
RbohF-F	AGTAAGCCTGGATACATAGAC	Pull down
RbohF-F	CAAGAAGGTGGTGTGAATAC	Pull down
35S-CAT1-F	CGCGTCGACATGGCAAGTG AAAAGTGGTT	Overexpression, RSS
35S-CAT1-R	TGCTCTAGATATGCTTGGTCTCACA	Overexpression, RSS
35S-CAT3-F	CGCGTCGACATGGATCCATACAAGTATCG	Overexpression, RSS
35S-CAT3-R	CGGGGTACCCATTGTGGGCCTTACAT	Overexpression, RSS
35S-HCPro-F	CGCGTCGACATGTCAGCAGGCGAGCTCTT	Overexpression, RSS
35S-HCPro-R	CGGGGTACCCTAACCAACTCTGTACATT	Overexpression, RSS
DT1- CAT1-BsF	ATATATGGTCTCGATTACAGGCATATGGACGGATCGTT	Knock out
DT1-CAT1-F0	TGACAGGCATATGGACGGATCGTTGTTTTAGAGCTAGAAATAGC	Knock out
DT2-CAT1-R0	ATATATGGTCTCGATTGATCCGTCCATATGCCTGTAAGAGTT	Knock out
DT2-CAT1-BsR	ATTATTGGTCTCGAAACGATCCGTCCATATGCCTGTAAGACAA	Knock out
DT1- CAT3-BsF	ATATATGGTCTCGATTGCAGATCTTGGGCACCGGACGTT	Knock out
DT1-CAT3-F0	TGCAGATCTTGGGCACCGGACGTTTTAGAGCTAGAAATAGC	Knock out
DT2-CAT3-R0	AACGTCCGGTGCCCAAGATCTGCAATCTCTTAGTCGACTCTAC	Knock out
DT2-CAT3-BsR	ATTATTGGTCTCGAAACGTCCGGTGCCCAAGATCTGCAA	Knock out
DT2-CAT3-R0	AACGAGGTGCCAGCGCCAAGGGCAATCTCTTAGTCGACTCTAC	Knock out
DT2-CAT3-BsR	ATTATTGGTCTCGAAACGAGGTGCCAGCGCCAAGGGCAA	Knock out
U626-IDF	TGTCCCAGGATTAGAATGATTAGGC	Knock out
U629-IDR	AGCCCTCTTCTTTCGATCCATCAAC	Knock out

Cas-CAT1-F	ACGTGTCCCT GAACGTGTTG	Knock out
Cas-CAT1-R	ACTTCACATAGGTTGATTTC	Knock out
Cas-CAT3-F1	CTTGGGCCAAACTATCTGCA	Knock out
Cas-CAT3-R1	GACGCAATCTTCTGACCAAG	Knock out
Cas-CAT3-F2	ATGGATCCATACAAGTATCGT	Knock out
Cas-CAT3-R2	GACGCAATCTTCTGACCAAG	Knock out
HCPro-GFP-F	CGCGTCGACATGTCAGCAGGCGAGCTCTT	Subcellular location
HCPro-GFP-R	CGCGGATCCCTAACCAACTCTGTACATT	Subcellular location
CAT1-mCherry-F	CCGCTCGAGATGGATCCATACAAGTACCG	Subcellular location
CAT1-mCherry-R	CGCGTCGACTATGCTTGGTCTCACA	Subcellular location
CAT3-mCherry-F	CCGCTCGAGATGGATCCATACAAGTATCG	Subcellular location
CAT3-mCherry-R	CGCGTCGACCATTGTGGGCCTTACAT	Subcellular location

Table S1. Primers used for construction of vectors and real-time PCR analysis.
