# Supplementary material for: Chilli veinal mottle virus HCPro interacts with catalase to facilitate virus infection in Nicotiana tabacum
Source: J Exp Bot. 2020 Jun 28;71(18):5656–68. doi: 10.1093/jxb/eraa304 (PMC7501817; doi:10.1093/jxb/eraa304)
Supplement: eraa304_suppl_Supplementary_Table_S1 [file eraa304_suppl_supplementary_table_s1.pdf]

Primer Names	Primer Sequences (5'-3')	Purpose
Yeast-CAT1-F	CCGGAATTC ATGGCAAGTG AAAAGTGGTT	Y2H
Yeast-CAT1-R	GCACTGCAGTTACTGATGGGTGACTTCTC	Y2H
Yeast-CAT2-F	CCGGAATTCATGGATCCCTCTAAGTTTCGA	Y2H
Yeast-CAT2-R	GCACTGCAGTCACATTGTAGGCTTTAAAG	Y2H
Yeast-CAT3-F	CCGGAATTCATGGATCCATACAAGTATCG	Y2H
Yeast-CAT3-R	CGCGTCGACTCACATTGTGGGCCTTACAT	Y2H
Yeast-HCPro -F	CCGGAATTC ATGTCAGCAGGCGAGCTCTT	Y2H
Yeast-HCPro -R	CGCGGATCCCTAACCAACTCTGTACATT	Y2H
MBP-CAT1-F	CCGGAATTC ATGGCAAGTG AAAAGTGGTT	Pull down
MBP-CAT1-R	GCACTGCAGTTACTGATGGGTGACTTCTC	Pull down
MBP-CAT2-F	CCGGAATTCATGGATCCCTCTAAGTTTCGA	Pull down
MBP-CAT2-R	GCACTGCAGTCACATTGTAGGCTTTAAAG	Pull down
MBP-CAT3-F	CCGGAATTCATGGATCCATACAAGTATCG	Pull down
MBP-CAT3-R	CGCGTCGACTCACATTGTGGGCCTTACAT	Pull down
GST-HCPro-F	CCGGAATTCATGTCAGCAGGCGAGCTCTT	Pull down
GST-HCPro-R	CGCGTCGACCTAACCAACTCTGTACATT	Pull down
GST-CAT1-F	CCGGAATTCATGGCAAGTG AAAAGTGGTT	Pull down
GST-CAT1-R	GCAGTCGACTTACTGATGGGTGACTTCTC	Pull down
GST-CAT3-F	CCGGAATTCATGGATCCATACAAGTATCG	Pull down
GST-CAT3-R	CGCGTCGACTCACATTGTGGGCCTTACAT	Pull down
MBP-HCPro-F	CCGGAATTCATGTCAGCAGGCGAGCTCTT	Pull down
MBP-HCPro-R	CGCGTCGACCTAACCAACTCTGTACATT	Pull down
MBP-HC-N-F	CCGGAATTCATGTCAGCAGGCGAGCTCTT	Pull down
MBP-HC-N-R	CGCGTCGACGCTTTTAAGTAATTGTACAGCA	Pull down

MBP-HC-F	CCGGAATTCATGCACTTTTTAAGCTTTAAA	Pull down
MBP-HC-R	CGCGTCGACCGATCCATCATCATAAGTTACA	Pull down
MBP-HC-C-F	CCGGAATTCATGCCAGTTTTGTCAGAATTCAA	Pull down
MBP-HC-C-R	CGCGGATCCCTAACCAACTCTGTACATT	Pull down
BiFC-CAT1 -F	TGCTCTAGAATGGATCCATACAAGTACCG	BiFC
BiFC-CAT1-R	CGCGTCGACTATGCTTGGTCTCACA	BiFC
BiFC-CAT2-F	TGCTCTAGAATGGATCCCTCTAAGTTTCG	BiFC
BiFC-CAT2-R	CGCGTCGACCATTGTAGGCTTTAAAG	BiFC
BiFC-CAT3-F	TGCTCTAGAATGGATCCATACAAGTATCG	BiFC
BiFC-CAT3-R	CGCGTCGACCATTGTGGGCCTTACAT	BiFC
BiFC-HCPro-F	CCGGAATTCATGTCAGCAGGCGAGCTCTT	BiFC
BiFC-HCPro-R	CGCGTCGACCTAACCAACTCTGTACATT	BiFC
CAT1-F	TCCACAAGATTACAGGCATA	qPCR
CAT1-R	AGCGGCAATAGAGTCATAG	qPCR
CAT2-F	CCAATTCCTTCTCGTGTCT	qPCR
CAT2-R	GTATCTGTCTTGCCTGTCA	qPCR
CAT3-F	AGGAGGAGCGAATCATAGT	qPCR
CAT3-R	TTCAATACCAAGCGACCAA	qPCR
EF1α-F	TGCTGTAACAAGATGGATGC	qPCR
EF1α-R	AGATGGGGACAAAGGGGATT	qPCR
Coat protein-F	AAACCCAGCCACAGTCTCGT	qPCR
Coat protein-R	ATCTCCGTCCATCATCACCC	qPCR
NbEF1α-F	AGCTTTACCTCCCAAGTCATC	qPCR
NbEF1α-R	AGAACGCCTGTCAATCTTGG	qPCR
GFP-F	ACATTATGGCAGACAAACAA	qPCR

GFP-R	TTACAAACTCAAGAAGCACC	qPCR
HCPro-F	ATGTCAGCAGGCGAGCTCTT	RT-PCR
HCPro-R	CTAACCAACTCTGTACATT	RT-PCR
RbohD-F	ACACGATCACATGGCTTCGAAAT	qPCR
RbohD-R	GTGTATTCCAACCCCAAGAGCA	qPCR
RbohF-F	AGTAAGCCTGGATACATAGAC	qPCR
RbohF-R	CAAGAAGGTGGTGTGAATAC	qPCR
35S-CAT1-F	CGCGTCGACATGGCAAGTG AAAAGTGGTT	Overexpression, RSS
35S-CAT1-R	TGCTCTAGATATGCTTGGTCTCACA	Overexpression, RSS
35S-CAT3-F	CGCGTCGACATGGATCCATACAAGTATCG	Overexpression, RSS
35S-CAT3-R	CGGGGTACCCATTGTGGGCCTTACAT	Overexpression, RSS
35S-HCPro-F	CGCGTCGACATGTCAGCAGGCGAGCTCTT	Overexpression, RSS
35S-HCPro-R	CGGGGTACCCTAACCAACTCTGTACATT	Overexpression, RSS
DT1- CAT1-BsF	ATATATGGTCTCGATTACAGGCATATGGACGGATCGTT	Knock out
DT1-CAT1-F0	TGACAGGCATATGGACGGATCGTTGTTTTAGAGCTAGAAATAGC	Knock out
DT2-CAT1-R0	ATATATGGTCTCGATTGATCCGTCCATATGCCTGTAAGAGTT	Knock out
DT2-CAT1-BsR	ATTATTGGTCTCGAAACGATCCGTCCATATGCCTGTAAGACAA	Knock out
DT1- CAT3-BsF	ATATATGGTCTCGATTGCAGATCTTGGGCACCGGACGTT	Knock out
DT1-CAT3-F0	TGCAGATCTTGGGCACCGGACGTTTTAGAGCTAGAAATAGC	Knock out
DT2-CAT3-R0	AACGTCCGGTGCCCAAGATCTGCAATCTCTTAGTCGACTCTAC	Knock out
DT2-CAT3-BsR	ATTATTGGTCTCGAAACGTCCGGTGCCCAAGATCTGCAA	Knock out
DT2-CAT3-R0	AACGAGGTGCCAGCGCCAAGGGCAATCTCTTAGTCGACTCTAC	Knock out
DT2-CAT3-BsR	ATTATTGGTCTCGAAACGAGGTGCCAGCGCCAAGGGCAA	Knock out
U626-IDF	TGTCCCAGGATTAGAATGATTAGGC	Knock out
U629-IDR	AGCCCTCTTCTTTCGATCCATCAAC	Knock out

Cas-CAT1-F	ACGTGTCCCT GAACGTGTTG	Knock out
Cas-CAT1-R	ACTTCACATAGGTTGATTTC	Knock out
Cas-CAT3-F1	CTTGGGCCAAACTATCTGCA	Knock out
Cas-CAT3-R1	GACGCAATCTTCTGACCAAG	Knock out
Cas-CAT3-F2	ATGGATCCATACAAGTATCGT	Knock out
Cas-CAT3-R2	GACGCAATCTTCTGACCAAG	Knock out
HCPro-GFP-F	CGCGTCGACATGTCAGCAGGCGAGCTCTT	Subcellular location
HCPro-GFP-R	CGCGGATCCCTAACCAACTCTGTACATT	Subcellular location
CAT1-mCherry-F	CCGCTCGAGATGGATCCATACAAGTACCG	Subcellular location
CAT1-mCherry-R	CGCGTCGACTATGCTTGGTCTCACA	Subcellular location
CAT3-mCherry-F	CCGCTCGAGATGGATCCATACAAGTATCG	Subcellular location
CAT3-mCherry-R	CGCGTCGACCATTGTGGGCCTTACAT	Subcellular location
